# Hypereosinophilia Associated With Antisynthetase Syndrome With Anti‐Ro52/PL12 Co‐Positivity: An Unusual Presentation

**DOI:** 10.1002/ccr3.72096

**Published:** 2026-03-04

**Authors:** Abeline Kapuczinski, Marc Léon, Camelia Rossi, Benjamin Bondue, Nathalie Demeulenaere

**Affiliations:** ^1^ Rheumatology Department CHU HELORA site Kennedy Mons Belgium; ^2^ Infectious Disease Department CHU HELORA site Kennedy Mons Belgium; ^3^ Pneumology Department Hôpital Erasme Bruxelles Belgium

**Keywords:** anti‐Ro52/PL12 co‐positivity, antisynthetase syndrome, hypereosinophilia, idiopathic inflammatory myopathies, interstitial lung disease

## Abstract

Hypereosinophilia is uncommon in ASS and this presentation makes diagnosis more challenging. Anti‐PL12's role in the severity of ILD is controversial but its association with anti‐Ro52 seems to give a more serious disease phenotype. African ethnicity could be a negative prognostic factor, associated with lung disease severity.

## Graphical abstract

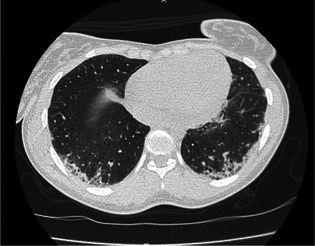

## 1 Introduction

Antisynthetase syndrome (ASS) is a subcategory of idiopathic inflammatory myopathies (IIMs) characterized by the presence of myositis‐specific autoantibodies (MSA) directed against aminoacyl‐tRNA‐synthetases (ARS) [1, 2]. ASS affects 11.1%–39.9% of patients with IIM with a female–male ratio approximately 7:3 and mean age at the beginning of the disease of 48 ± 15 years [1].

To date, autoantibodies to ten ARS have been determined. Among them, anti‐Jo‐1 is the most common, followed by anti‐EJ, anti‐PL7, anti‐PL12, and anti‐OJ [3]. ASS pathogenesis is still not fully explained. The disease seems to begin in the lung after pulmonary aggression by environmental factors and/or infectious agents, leading to unspecific immune stimulation, generation of neo‐antigens, a tolerance breakdown with generation of specific T and B cells, and finally disease propagation to target tissues resulting in organ damage [2].

Clinical presentation of ASS includes myositis, arthritis, interstitial lung disease (ILD), Raynaud's Phenomenon (RP), fever and cutaneous manifestations like Mechanic's hands. The characteristic “triad” with arthritis, myositis, and ILD is observed in up to 90% of cases [4]. ILD is a key feature of ASS, with a prevalence between 70% and 95% in ASS patients depending on the cohort, leading to high mortality and morbidity [4].

There is a striking heterogeneity among different anti‐ARS antibodies in prevalence and severity of ILD. Anti‐Jo‐1 antibody seems to be related to lower rates of ILD but higher rates of myositis and joint involvement while patients with anti‐PL7 and anti‐PL12 autoantibodies are more likely to have ILD [4, 5]. In particular, Anti‐PL12 has been associated with less myositis presentation but rapidly progressive ILD [5]. Anti‐Ro52, a myositis‐associated antibody found in polymyositis/dermatomyositis patients, has been linked with increased severity of ILD, more severe muscle and joint involvement, and higher malignancy when co‐existing with anti‐Jo‐1 [5]. Rapidly progressive ILD arises more in the non‐anti‐Jo‐1 patients, especially in those with co‐existing anti‐PL7 and anti‐Ro52, while Anti‐PL12 and anti‐Ro52 co‐positivity is not well established [4, 5].

Association with hypereosinophilia is not really described in previous studies and case reports. We only found, up to now, two case reports mentioning hypereosinophilia: one with eosinophilic pneumonia preceding ASS [6] and the other ASS with eosinophilic pleural effusion [7]. Relation between both entities is unclear.

We here describe the case of a young African female, having myositis and ILD as part of ASS with anti‐PL12 and anti‐Ro52 co‐positivity and presenting with hypereosinophilia.

## 2 Case History

A 17‐year‐old African patient was admitted to the Emergency Department (ED) because of ramping arthralgia for a month and recent dyspnea. She is of Congolese origin, born in Belgium and she did not travel recently. She had never smoked and had a medical history of asthma. She is not taking any medication. She had no fever. Admission physical examination did not show any swollen joints or cutaneous lesions but pulmonary auscultation revealed lung crackles. Lymph nodes examination was normal. She was admitted to the infectious disease/internal medicine department for more investigations.

## 3 Methods: Differential Diagnosis, Investigations and Treatment

Laboratory tests revealed high creatine phosphokinase (CPK) level (1639 UI/L; normal range 29.0–168.0 UI/L), elevated erythrocyte sedimentation rate (27 mm/1st h; normal < 15) and C‐reactive protein (24 mg/L; normal < 5) with hypereosinophilia (4650/μL; normal range 20–600/μL), elevated lactate dehydrogenase (644 UI/L; normal range 125.0–220.0 UI/L) and GOT (57 U/L; normal range 5.0–34.0 U/L) levels. Further investigations showed that the patient was positive for the presence of antinuclear antibodies (ANA), titer 1:640 with anti‐Ro52 and anti‐PL12 identification and negative for the presence of anti‐neutrophil cytoplasmic antibodies (ANCA). Rheumatoid factor (RF) and anti‐CCP were also negative.

Parasitic infections and allergy were excluded (parasitic serologies for trichinella, toxocara, ascaris, strongyloides, fasciola hepatica were negative, as was the stool examination for parasites; Immunoglobulin E (IgE) level was not significant, no medication could explain eosinophilia and normal blood immunophenotyping ruled out idiopathic hypereosinophilic syndrome. There were no other clinical features consistent with Sjögren's syndrome (no sicca symptoms), nor with systemic lupus erythematosus (no photosensitivity, no other biological anomalies), nor rheumatoid arthritis (no clinical arthritis and RF and anti‐CCP negativity), nor with vasculitis.

**FIGURE 1:**
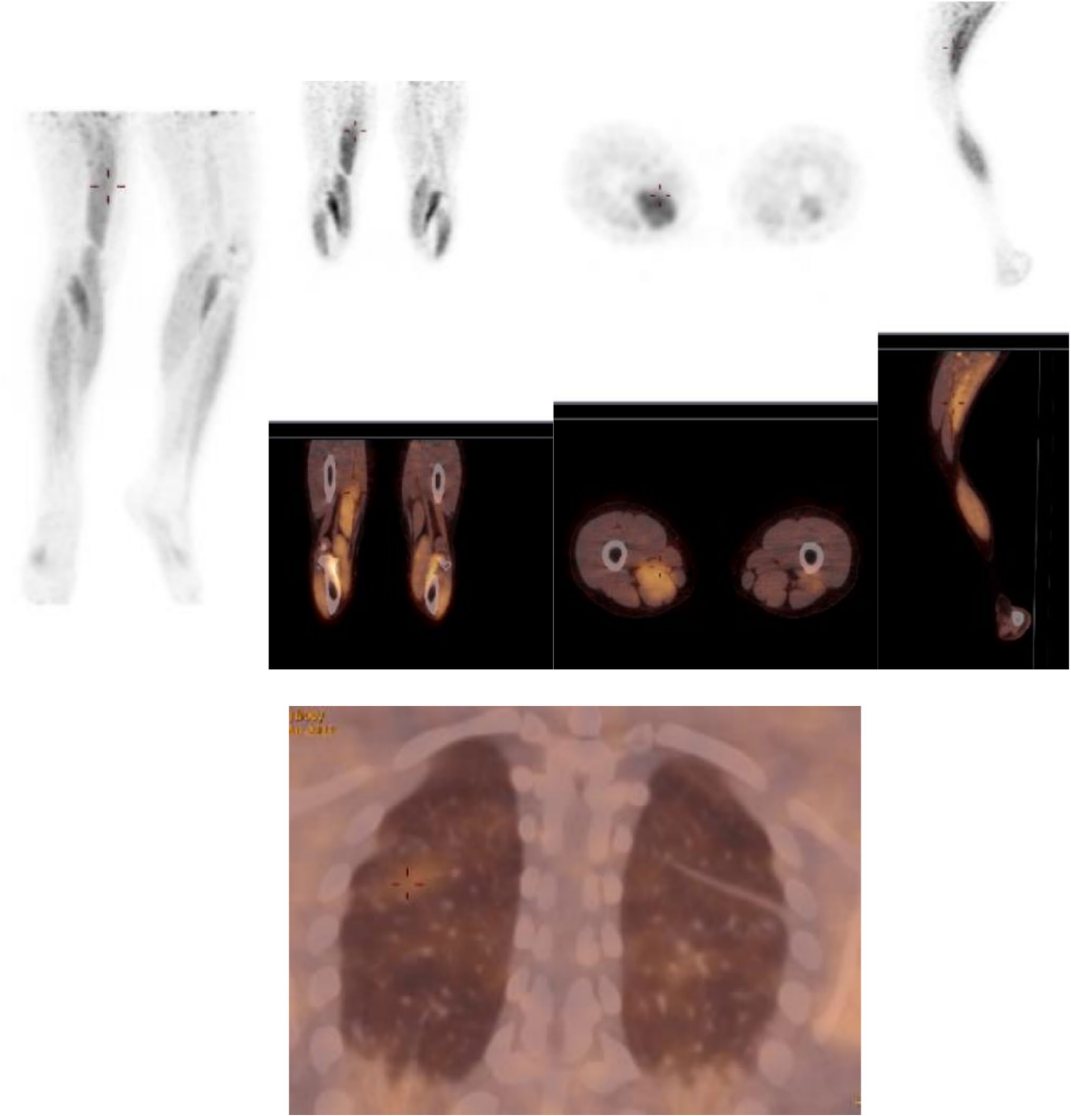
PET‐CT scan showing scapular and legs muscle hypermetabolism.

**FIGURE 2:**
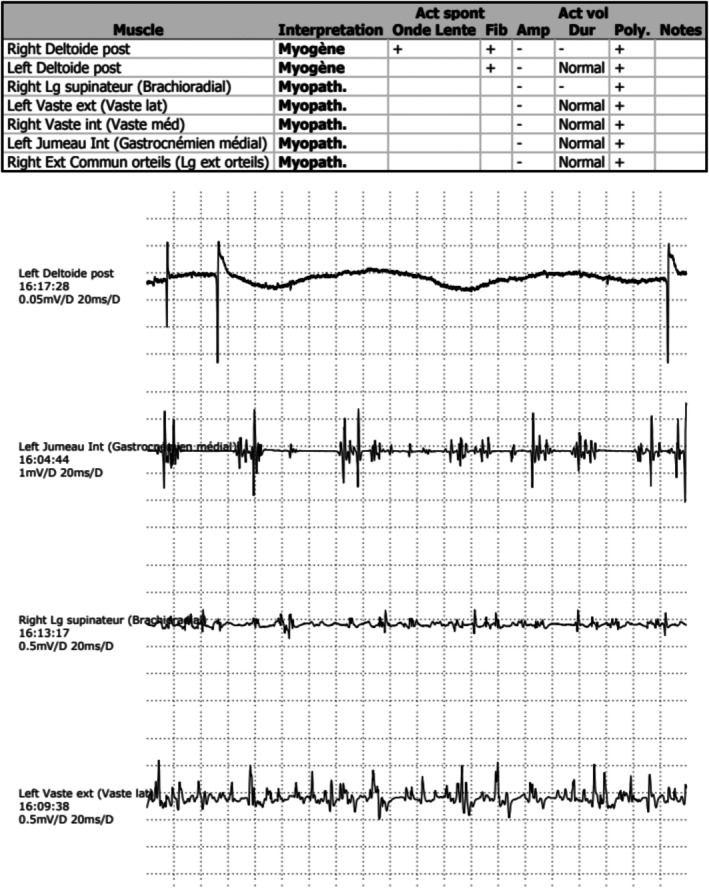
Detailed electromyography (EMG) findings. Report showing some anomalies of a myopathic nature in the various muscles investigated, as well as more myositic anomalies in the left and right deltoids (myopathic potentials + fibrillations and positive sharp wave).

**FIGURE 3:**
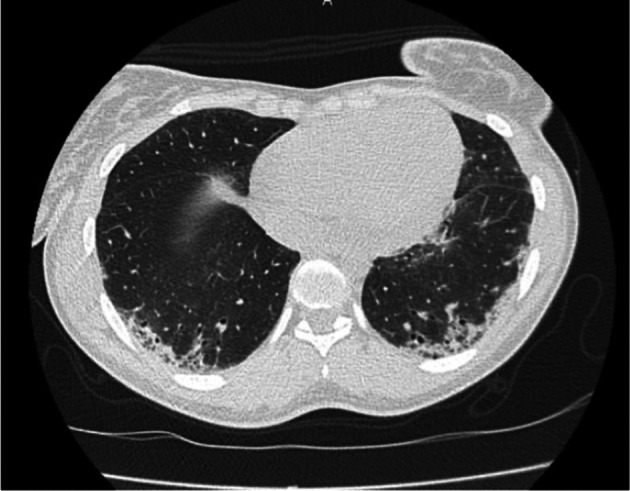
Chest computed tomography showing ground glass opacities suggestive of interstitial pneumonia at pulmonary bases with bronchiectasis.

PET‐CT scan showed scapular and legs muscle hypermetabolism (Figure 1). Electromyogram was consistent with myositis, report showing some anomalies of a myopathic nature in the various muscles investigated, as well as more myositic anomalies in the left and right deltoids (myopathic potentials, fibrillations and positive sharp wave) (Figure 2). Chest computed tomography identified ground glass opacities suggestive of interstitial pneumonia associated with connective tissue disease (Figure 3). Pulmonary function test showed a restrictive lung disease with a reduction in carbon monoxide diffusion capacity (DLCO) of 41%, total lung capacity (TLC) of 61%, and forced vital capacity (FVC) of 58% of predictive values.

Immunosuppressive therapy with pulse methylprednisolone (mPDN) 1 mg/kg/day for 3 days was started, and continued with progressively reduced doses while mycophenolate mofetil (MMF) as a steroid‐sparing immunosuppressant up to 2 g/day was added. This treatment was discussed in a multi‐disciplinary way according to the actual guidelines (see discussion). Pulse corticotherapy is the first line of treatment. MMF was selected in light of the patient's individual profile and due to its favorable safety profile, which does not necessitate intensive monitoring.

## 4 Conclusion and Results: Outcome and Follow‐Up

**FIGURE 4:**
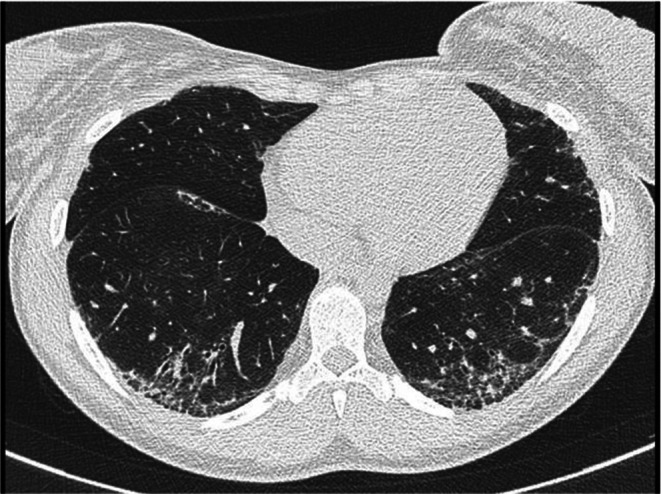
Chest computed tomography control at 9‐month follow‐up showing improvement of ground glass opacities associated with lesions suggestive of fibrosis.

**FIGURE 5:**
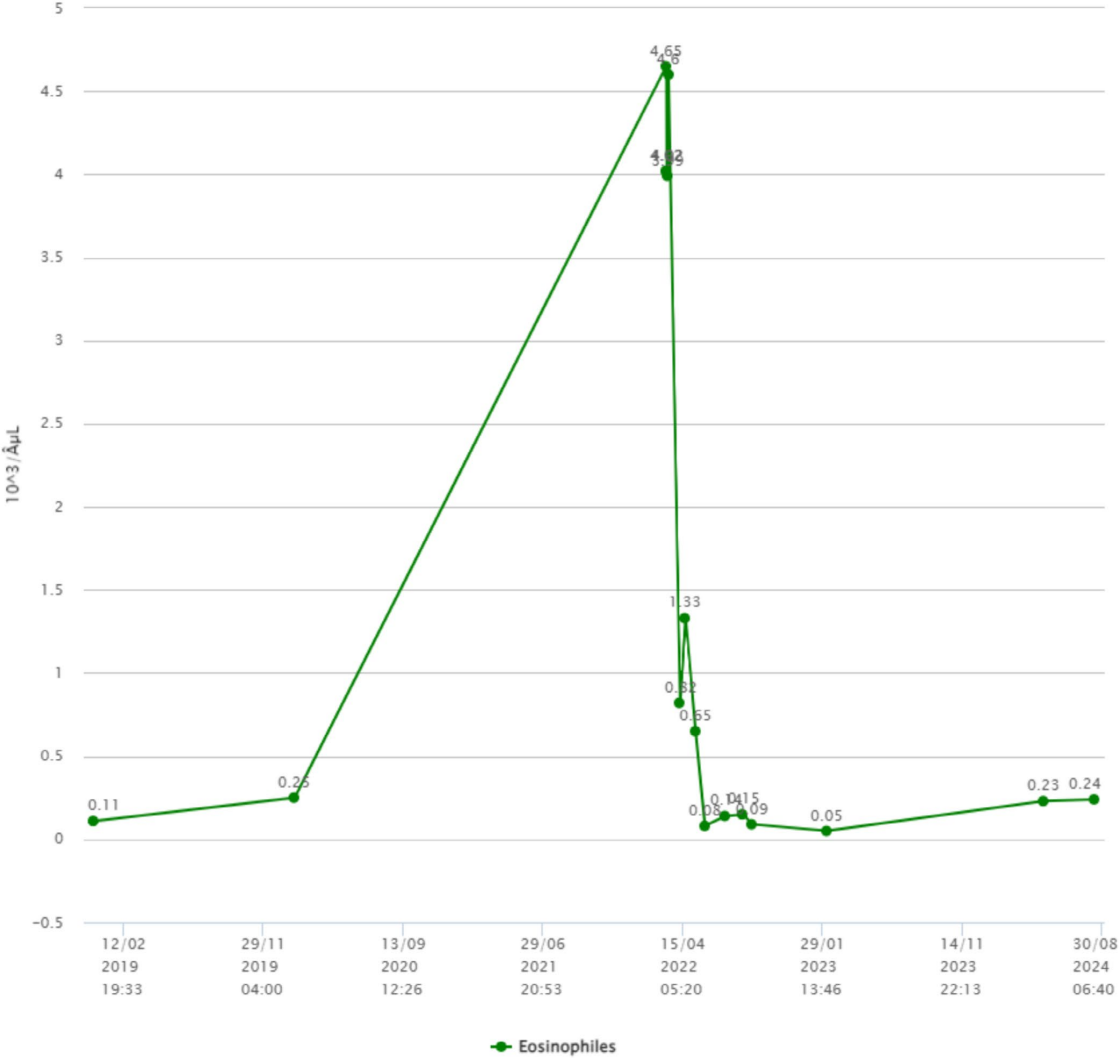
Evolution graph of eosinophils before (2019 to 2021), during (2022), and after (2022 to 2024) treatment.

**FIGURE 6:**
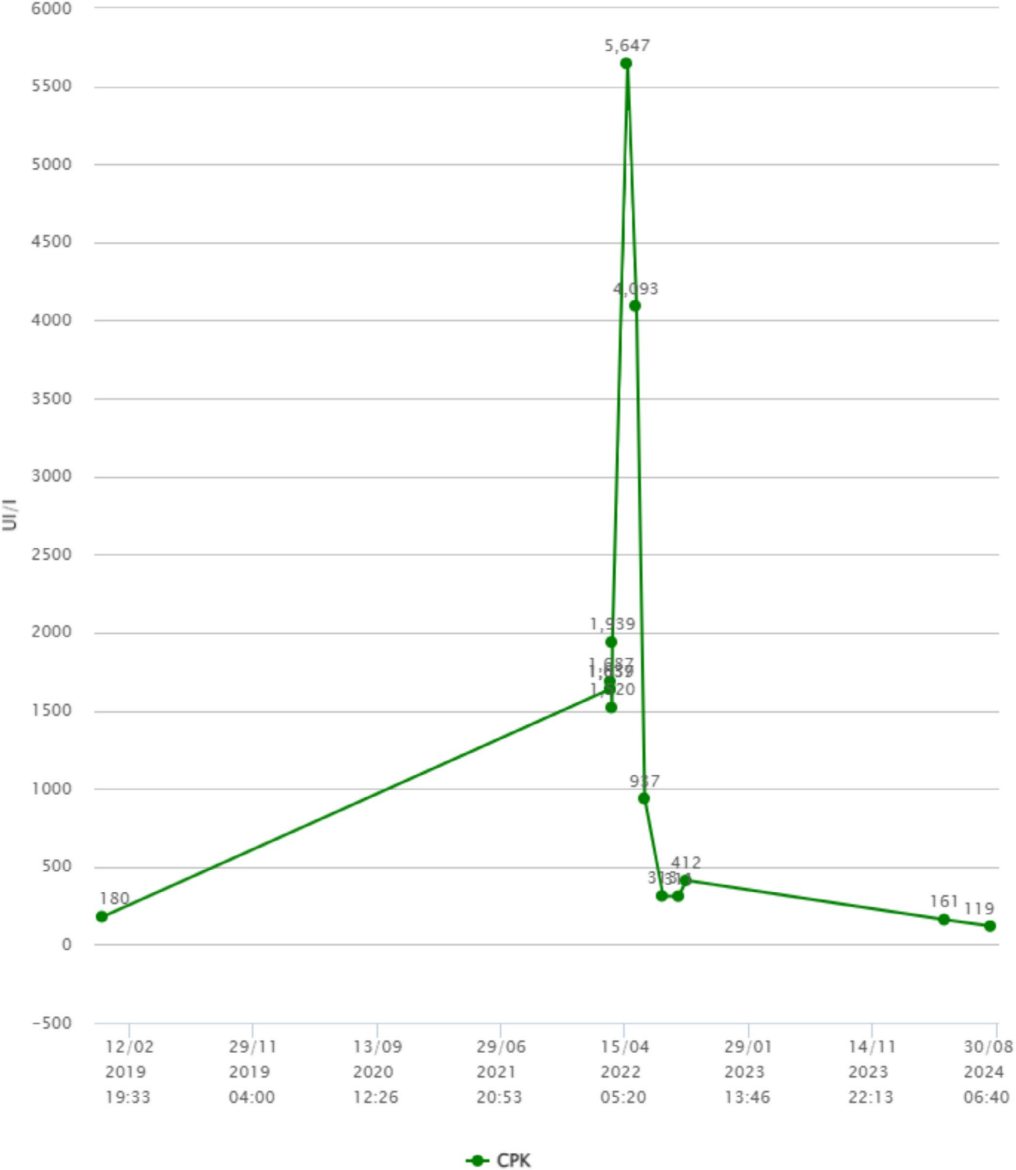
Evolution graph of CPK levels before (2019 to 2021), during (2022), and after (2022 to 2024) treatment.

Antisynthetase syndrome diagnosis was established. Hypereosinophilia was attributed to auto‐immune context. At 6‐month follow up, the patient no longer had arthralgia, muscular pain, or dyspnea. A control chest CT scan at 9‐month follow up showed improvement of ground glass opacities associated with lesions suggestive of fibrosis (Figure 4), increased FVC at 77% and DLCO of 56% of predictive values. Eosinophilia and CPK levels returned to normal values (Figures 5 and 6). At 2‐year follow up, mPDN was stopped without relapse and with a stable state of the disease. Medication with MMF 2 g/day is still ongoing.

## 5 Discussion

ASS is a rare autoimmune disease, which is important to diagnose because of its life threatening nature related to severe lung involvement.

Interestingly, our patient presented with hypereosinophilia as one of the early findings which are not classical of ASS. We excluded common causes of eosinophilia and attributed it to the auto‐immune context and probably the presence of eosinophilic pneumonia. Bronchoalveolar lavage was not performed because of the evidence of ASS disease and the rapidly recovering eosinophil normal values after treatment. We only found two case reports about eosinophilia with ASS: Saito et al. describing eosinophilic pleural effusion [7] and Hachisu et al. reporting the onset of eosinophilic pneumonia preceding ASS [6].

ASS is characterized by the presence of specific antisynthetase antibodies, which seem to influence the clinical presentation, evolution and prognosis. According to several studies, anti‐Jo‐1 antibodies seem to be associated with higher rates of myositis and joint involvement while patients with anti‐PL7 and anti‐PL12 autoantibodies have more frequently ILD [8]. Marie et al. showed that the presence of anti‐Jo‐1 antibody results in more severe myositis, joint involvement and increased risk of cancer while anti‐PL7/PL12 antibodies were associated with early and severe ILD; so their findings described some differences in clinical phenotype and disease evolution [9]. However, other studies did not find a significant difference between patients positive for anti‐Jo‐1 and patients positive for non‐anti‐Jo‐1 antibodies. Dei et al. confirmed in their study the absence of distinction in pulmonary functional disease evolution in patients with anti‐Jo‐1 versus non anti‐Jo‐1 [3]. Cavagna et al. showed that the clinical expression and evolution of anti‐Jo‐1, PL7, PL12, EJ and anti‐OJ positivity in ASS was similar, pointing out that ASS is a heterogeneous condition and that antibody specificity only partly influences the clinical picture and outcome of this situation [10].

Anti‐SSA/Ro52 antibodies can be found in about 30%–50% of patients with ASS [3]. La Corte et al. showed that the association of anti‐Jo‐1 and anti‐Ro/SSA antibodies seems to give more severe ILD [11]. In their study, Dei et al. observed that anti‐SSA/Ro52 positivity was more prevalent in non‐anti‐Jo‐1 patients [3]. Anti‐PL12 and anti‐SSA/Ro52 co‐positivity detected in our patient is not well described but anti‐PL12 phenotypic presentation associated with anti‐SSA/Ro52 may give a more aggressive disease with greater muscle involvement and increased severity of ILD [5].

We also mentioned in our case report that our patient was of Congolese origin. Pinal‐Fernandez et al. described in their work that the different antisynthetase autoantibodies were related to phenotypically distinct subtypes within the antisynthetase spectrum, with anti‐PL7 and anti‐PL12 antibodies associated with more aggressive ILD. Their study also showed that African ethnicity was a major prognostic factor associated with lung disease severity [12].

ASS is a heterogeneous disease without specific established classification criteria. To date, ASS takes part of IIMs even if myositis is not always the main manifestation. Some new classifications and diagnostic criteria of IIM and its subtypes have been suggested, like Solomon's criteria and Conor's criteria [1]. Because it is a heterogeneous condition, ASS diagnosis may be challenging as patients are sometimes diagnosed with undifferentiated connective tissue disease (UCTD), idiopathic ILD, interstitial pneumonia with autoimmune feature (IPAF), or other connective tissue disease (CTD) [1, 13]. More studies are needed to provide specific classification criteria for ASS.

Management of ASS is discussed because of the lack of studies. Treatment recommendations based on trials, retrospective analyses and expert opinion consist of corticosteroids in the first line therapy but long‐term use is limited by significant side effects [14]. Other immunosuppressive agents like azathioprine (AZA), MMF or cyclophosphamide are considered in more severe disease and particularly in patients with ILD who are at risk of poor response to corticosteroid monotherapy. Both AZA and MMF proved to be efficacious in IIM‐associated ILD. AZA and methotrexate (MTX) are commonly used steroid‐sparing agents, with MTX acting more rapidly but contraindicated in severe pulmonary involvement. MMF is favored for interstitial lung disease due to its selective lymphocyte inhibition and better tolerability. Intravenous immunoglobulins and calcineurin inhibitors (e.g., tacrolimus, cyclosporine) are considered in refractory cases [14]. Cyclophosphamide is also considered in refractory disease as rescue therapy but have many side effects including malignancies [14]. Biological agents such as Rituximab seems to be a promising therapeutic option in refractory cases [1, 14]. We decided in our case to treat with corticosteroids and MMF with successful improvement.

The main potential complication here would be linked to lung damage due to the severity of ILD (see discussion about severity of ILD in anti‐Ro52/PL12 co‐positivity [5]). Pulmonary hypertension due to ILD is another potential complication [14]. Fortunately, our patient did not develop any complications.

In conclusion, eosinophilia is uncommon in ASS presentation. Rare cases of eosinophilic pneumonia or eosinophilic pleural effusion have been described. Anti‐PL12 may be responsible for more severe ILD (it is controversial) whereas anti‐Ro52 seems to be related to earlier onset of arthritis, Mechanic's hands and dermatomyositis‐specific skin findings. Co‐existence with anti‐Ro52 may give a more serious disease phenotype, in particular more severe ILD. Moreover, African ethnicity could be a negative prognostic factor, associated with lung disease severity. Early diagnosis is essential and immunosuppressive treatment is the cornerstone for recovery. Due to ASS disease heterogeneity, more studies are needed to establish treatment recommendations and more specific classification criteria.

## Author Contributions

**Abeline Kapuczinski:** conceptualization, writing – original draft, writing – review and editing. **Marc Léon:** supervision. **Camelia Rossi:** supervision. **Benjamin Bondue:** supervision. **Nathalie Demeulenaere:** supervision.

## Funding

The authors have nothing to report.

## Consent

Written informed consent has been taken from the patient.

## Data Availability

Data available on request from the authors.
